# Multiday Use of Non‐Tunneled Central Venous Catheters in Vetted Adult Outpatients Undergoing Apheresis Collections of Autologous Peripheral Blood Stem Cells Is Feasible and Well Tolerated

**DOI:** 10.1002/jca.70120

**Published:** 2026-03-31

**Authors:** Andres E. Mindiola Romero, Joseph R. Griggs, Marian A. Rollins‐Raval, Matthew L. Fero, Jay S. Raval

**Affiliations:** ^1^ Department of Pathology University of New Mexico Health Sciences Center Albuquerque New Mexico USA; ^2^ Department of Pathology and Laboratory Medicine, Robert Larner, M.D. College of Medicine University of Vermont Burlington Vermont USA; ^3^ Department of Internal Medicine University of New Mexico Health Sciences Center Albuquerque New Mexico USA

**Keywords:** adverse event, mononuclear cell collection, non‐tunneled catheter, outpatient, stem cell collection, untunneled catheter

## Abstract

Autologous peripheral blood stem cell (PBSC) collections are routinely performed in the outpatient setting and typically involve placement of an apheresis‐compatible central venous catheter (CVC). There is reluctance to discharge outpatients with non‐tunneled CVCs due to safety concerns. We characterized our center's practice of multiday non‐tunneled CVC use in vetted adult outpatients undergoing autologous PBSC collections. No patient with social, cognitive, hygienic, caregiver, or bleeding concerns had non‐tunneled CVC placement. All patients and caregivers were counseled about potential risks of non‐tunneled CVCs and were provided with basic CVC care education. In total, 117 autologous PBSC collections were performed in 65 adult outpatients via non‐tunneled CVCs. Apheresis nursing staff removed CVCs from all patients on their final PBSC collection day. There was one minor CVC site bleed that was successfully controlled with manual compression. In our small population, multiday non‐tunneled CVC use in vetted adult outpatients undergoing autologous PBSC collections was feasible and well tolerated.

Autologous peripheral blood stem cell (PBSC) collections are routinely performed to treat a variety of hematologic and oncologic disorders. Vascular access options for these apheresis collections include peripheral veins or a central venous catheter (CVC). However, there is reluctance to discharge outpatients from the healthcare setting with non‐tunneled CVCs due to rare clinical concerns of significant bleeding from the catheter site, accidental removal or repositioning, air embolism, line damage, infection, and local tissue injury [1, 2]. Herein, we characterize the feasibility and tolerability of multiday non‐tunneled CVC use in vetted adult outpatients undergoing autologous PBSC collections.

We conducted a retrospective study at our university medical center and comprehensive cancer center of autologous PBSC collections via non‐tunneled CVCs in adult outpatients performed between 2018 and 2023. These patients were identified through apheresis procedure logs, and a full chart review from initial transplant consultation to PBSC infusion was performed for every patient. No patient with social, cognitive, hygienic, caregiver, or bleeding concerns had non‐tunneled CVC placement [3, 4]. All patients and caregivers were counseled about the potential risks of having a non‐tunneled CVC and were provided education regarding precautions and basic CVC care in the outpatient setting. All patients had their non‐tunneled CVCs successfully placed at least 1 day prior to their apheresis PBSC collections. Notably, all patients had their overnight stays close to our institution and near emergency care services. CVC assessment and care was performed by the apheresis team every day. Data collected included patient demographics, disease indication, number of apheresis procedures, and CVC‐associated details including number of catheter days and complications such as bleeding from the catheter site, accidental catheter removal or repositioning, air embolism, line damage, infection, and local tissue injury. Descriptive data is presented as mean (standard deviation) or number (percentage).

In the 6‐year period, 117 autologous PBSC collections were performed in 65 adult outpatients via non‐tunneled CVCs (see Table 1); all were cannulated in an internal jugular vein. Overall, there were 65 catheter insertions (i.e., 1 per patient) and 194 total CVC days. Apheresis PBSC collections were performed using the Spectra Optia Apheresis System (Terumo BCT, Lakewood, CO, USA) and combination citrate/unfractionated heparin anticoagulation was employed. The maximum volume of patient blood processed during any apheresis PSBC collection procedure was either 30 L or 6 total blood volumes (whichever was lesser) and was modified by use of our CD34 collection algorithm once daily peripheral blood CD34 counts had resulted [5, 6]. The average duration of non‐tunneled CVC days in these outpatients was 3.0 (0.9) days. The only observed complication was a mild grade 1 bleeding event from a patient's CVC site that was successfully controlled with application of manual pressure only (bleeding rate per procedure = 1/117, 0.85%, 95% CI < 0.0001–0.0516; bleeding rate per patient = 1/65, 1.54%, 95% CI < 0.0001–0.0900) [7, 8]. This occurred at the patient's home and instructions regarding CVC care were followed by the caregiver (including notification of the event and its resolution to the on‐call transplant nurse). No other documented CVC‐associated complications were identified. The apheresis nursing staff successfully removed CVCs from all patients on the final apheresis PBSC collection day without issues.

**TABLE 1:** Patient demographics and PBSC collection characteristics.

Patient	Age (years)	Sex	Race	Ethnicity	Diagnosis	PBSC collections	CVC days
65	58.8 (10.5)	37 (56.9%) Male	50 (76.9%) White	39 (60%) Not Hispanic/Latino	55 (84.6%) Multiple myeloma	1.8 (0.8)	3.0 (0.9)

*Note:* Descriptive data is presented as mean (standard deviation) or number (percentage).

Abbreviations: CVC, central venous catheter; PBSC, peripheral blood stem cell.

Multiday non‐tunneled CVC use in vetted adult outpatients undergoing autologous apheresis PBSC collections was feasible and well tolerated in our small population. This practice allowed apheresis PBSC collections to extend over several days, if necessary, without the need for a more invasive tunneled CVC to be placed, as well as facilitated easier vascular access line removals since non‐tunneled CVCs were removed immediately at the bedside after the final apheresis PBSC collections. The initial impetus for this practice was to enhance patient comfort and satisfaction via a more rapid and less invasive non‐tunneled CVC placement and removal process, though decreased outpatient procedure costs were attendant benefits. However, during the COVID‐19 pandemic with critical hospital census levels of up to 150% capacity necessitating crisis standards of care and need for hospital beds and resources [9], strategies such as ours that minimized use of hospital specialty services were highly encouraged and further supported continuation of this practice. Observed bleeding rates in the current analysis were similar to the low rates associated with PBSC collection reported in large registry analyses [10, 11], as well as in patients at our institution deemed to have low bleeding risk who received up to 5 consecutive daily TPE using exclusively albumin fluid replacement without empiric fibrinogen testing or fibrinogen supplementation (bleeding rates were all single digit percentages) [12]. No infections or thromboses were observed. It is unknown if such an outpatient non‐tunneled CVC strategy could be expanded to other patient populations including children, though this may be possible based on previous successful inpatient experiences [13, 14].

We acknowledge that the current analysis is based on a small population with relatively few CVC days compared to studies that investigate CVC complication rates. However, most of these studies generate conclusions based on data from heterogeneous inpatients in high‐acuity healthcare settings and focus on either the very acute complications associated with CVC insertions or those with longer latency periods such as central line‐associated bloodstream infection (CLABSI) or thrombosis [2]. The current analysis is different in that our assessment of non‐tunneled CVCs focuses on investigating adverse events associated with a unique patient location, that is, the outpatient setting, over a brief period of CVC days, that is, an average of a few days, and in a unique patient population, that is, autologous PBSC donors.

It must be noted that CVCs are specifically designed and intended for short‐term, temporary use and, when appropriately used, have an excellent safety and efficacy profile [15]. Vascular access guidelines have discouraged the use of non‐tunneled CVCs in the outpatient setting due to the need for special surveillance and risks of complications [16, 17]; however, these guidelines are often focused on non‐vetted patient populations with conditions requiring longer‐term vascular access needs. Admittedly, these guidelines are not universally adhered to, and the short‐term complication differential between non‐tunneled CVCs and tunneled CVCs may not be as considerable as once thought [18]. In one recent analysis of acute hemodialysis patients, non‐tunneled CVCs did not significantly differ in function or safety from tunneled CVCs within 10 days of placement [19]. While the study did not specify inpatient versus outpatient status, a subset of patients were reported to require hospitalization to address CVC‐associated infections, thus suggesting use of non‐tunneled CVCs in the outpatient setting. In an international survey of sites performing PBSC collections, over half of the 80 respondents performing autologous PBSC collections reported that non‐tunneled CVCs were placed 1 or more days prior to the apheresis procedure [20]. While this survey did not comment on whether these procedures were performed in the inpatient or outpatient setting, the findings confirm that there are institutions keeping non‐tunneled CVCs in these patients for multiple days.

Patients undergoing autologous PBSC collections represent a unique population that is distinct from other patient groups requiring vascular access solutions. We assert that our practice positively capitalizes on the benefits of short‐term non‐tunneled CVC use for those undergoing autologous PBSC collection while also providing the necessary longitudinal assessments and care to minimize adverse events in the outpatient setting. Exploration and characterization of different approaches such as ours is supported by recent messaging from vascular access guideline groups about the need to move away from a “one‐size‐fits‐all” approach to strategies that provide more individualized care and balance patient goals and preferences with quality and safety so that we get closer to achieving the overarching goal of placing “the right access in the right patient, at the right time, for the right reasons” [16, 18]. Future, larger analyses performing prospective monitoring of this strategy utilizing non‐tunneled CVCs in outpatient autologous PBSC donors with a more prescriptive vetting plan will assist in characterizing generalizability and capturing additional low‐frequency events.

## Funding

The authors have nothing to report.

## Disclosure

The authors have nothing to report.

## Conflicts of Interest

The authors declare no conflicts of interest.

## Data Availability

The data that support the findings of this study are available from the corresponding author upon reasonable request.

## References

[1] E. Clark , J. Kappel , J. MacRae , et al., “Practical Aspects of Nontunneled and Tunneled Hemodialysis Catheters,” Canadian Journal of Kidney Health and Disease 27, no. 3 (2016): 2054358116669128. 10.1177/2054358116669128 PMC5332079 28270920

[2] B. Teja , N. A. Bosch , C. Diep , et al., “Complication Rates of Central Venous Catheters: A Systematic Review and Meta‐Analysis,” JAMA Internal Medicine 184, no. 5 (2024): 474–482. 38436976 10.1001/jamainternmed.2023.8232 PMC12285596

[3] Y. L. Chee , J. C. Crawford , H. G. Watson , and M. Greaves , “Guidelines on the Assessment of Bleeding Risk Prior to Surgery or Invasive Procedures. British Committee for Standards in Haematology,” British Journal of Haematology 140, no. 5 (2008): 496–504. 18275427 10.1111/j.1365-2141.2007.06968.x

[4] W. Lester , C. Bent , R. Alikhan , et al., “A British Society for Haematology Guideline on the Assessment and Management of Bleeding Risk Prior to Invasive Procedures,” British Journal of Haematology 204, no. 5 (2024): 1697–1713. 38517351 10.1111/bjh.19360

[5] E. R. Rosenbaum , B. O'Connell , and M. Cottler‐Fox , “Validation of a Formula for Predicting Daily CD34(+) Cell Collection by Leukapheresis,” Cytotherapy 14, no. 4 (2012): 461–466. 22277012 10.3109/14653249.2011.652733

[6] A. F. Cousins , J. E. Sinclair , M. J. Alcorn , et al., “HPC‐A Dose Prediction on the Optia Cell Separator Based on a Benchmark CE2 Collection Efficiency: Promoting Clinical Efficiency, Minimizing Toxicity, and Allowing Quality Control,” Journal of Clinical Apheresis 30 (2015): 321–328. 25619791 10.1002/jca.21380

[7] K. Zeidler , K. Arn , O. Senn , et al., “Optimal Preprocedural Platelet Transfusion Threshold for Central Venous Catheter Insertions in Patients With Thrombocytopenia,” Transfusion 51, no. 11 (2011): 2269–2276. 21517892 10.1111/j.1537-2995.2011.03147.x

[8] F. L. Van Baarle , E. K. Van De Weerdt , V. Der Velden , et al., “Platelet Transfusion Before CVC Placement in Patients With Thrombocytopenia,” New England Journal of Medicine 388, no. 21 (2023): 1956–1965. 37224197 10.1056/NEJMoa2214322

[9] Medical Advisory Team , “New Mexico Department of Health,” Supplement of the New Mexico Crisis Standards of Care Plan, Available from, https://cv.nmhealth.org/wp‐content/uploads/2020/05/NM‐CSC‐Plan‐V.5.1.20.pdf.

[10] M. Mörtzell Henriksson , E. Newman , V. Witt , et al., “Adverse Events in Apheresis: An Update of the WAA Registry Data,” Transfusion and Apheresis Science 54, no. 1 (2016): 2–15. 26776481 10.1016/j.transci.2016.01.003

[11] H. Vrielink , K. Le Poole , B. Stegmayr , et al., “The World Apheresis Association Registry, 2023 Update,” Transfusion and Apheresis Science 62, no. 6 (2023): 103831. 37827962 10.1016/j.transci.2023.103831

[12] J. S. Raval , J. R. Griggs , A. Mindiola Romero , et al., “Multiple Consecutive Daily Therapeutic Plasma Exchange Using Exclusively Albumin Replacement Fluid in Low Bleeding Risk Patients is Associated with Only Rare and Mild Bleeds,” Transfusion (2026), 10.1111/trf.70159. 41872698

[13] L. Madero , D. Ruano , M. Villa , et al., “Non‐Tunneled Catheters in Children Undergoing Bone Marrow Transplantation,” Bone Marrow Transplantation 17, no. 1 (1996): 87–89. 8673061

[14] L. Madero , M. A. Díaz , A. Benito , et al., “Non‐Tunneled Catheters for the Collection and Transplantation of Peripheral Blood Stem Cells in Children,” Bone Marrow Transplantation 20, no. 1 (1997): 53–56. 9232257 10.1038/sj.bmt.1700841

[15] T. S. Ipe and M. B. Marques , “Vascular Access for Therapeutic Plasma Exchange,” Transfusion 58, no. Suppl 1 (2018): 580–589. 29443413 10.1111/trf.14479

[16] C. E. Lok , T. S. Huber , T. Lee , et al., “KDOQI Clinical Practice Guideline for Vascular Access: 2019 Update,” American Journal of Kidney Diseases 75 (2020): S1–S164. 32778223 10.1053/j.ajkd.2019.12.001

[17] GAVeCeLT , “GAVeCeLT Recommendations 2024 for the Indication, Implantation, and Management of Vascular Access Devices,” ed. M. Pittiruti and G. Scoppettuolo (2024), https://www.gavecelt.it/nuovo/sites/default/files/uploads/raccomandazioni‐gavecelt‐2024.pdf.

[18] C. T. Chan , P. J. Blankestijn , L. M. Dember , et al., “Dialysis Initiation, Modality Choice, Access, and Prescription: Conclusions From a Kidney Disease: Improving Global Outcomes (KDIGO) Controversies Conference,” Kidney International 96 (2019): 37–47. 30987837 10.1016/j.kint.2019.01.017

[19] N. Morisi , M. Montani , E. N. Ehode , et al., “Evaluating Short‐Term Outcomes of Tunneled and Non‐Tunneled Central Venous Catheters in Hemodialysis,” Journal of Clinical Medicine 13, no. 13 (2024): 3664. 38999230 10.3390/jcm13133664 PMC11242506

[20] M. F. O'Leary , N. M. Dunbar , H. C. Kim , et al., “Venous Access for Hematopoietic Progenitor Cell Collection: An International Survey by the ASFA HPC Donor Subcommittee,” Journal of Clinical Apheresis 31, no. 6 (2016): 529–534. 26762291 10.1002/jca.21445

